# A practical guide to adopting Bayesian analyses in clinical research

**DOI:** 10.1017/cts.2023.689

**Published:** 2023-12-07

**Authors:** Lauren B. Gunn-Sandell, Edward J. Bedrick, Jacob L. Hutchins, Aaron A. Berg, Alexander M. Kaizer, Nichole E. Carlson

**Affiliations:** 1 Department of Biostatistics and Informatics, Colorado School of Public Health, Aurora, CO, USA; 2 Center for Innovative Design and Analysis, Colorado School of Public Health and University of Colorado School of Medicine, Aurora, CO, USA; 3 Department of Epidemiology and Biostatistics, University of Arizona, Tuscon, AZ, USA; 4 Department of Anesthesiology, University of Minnesota, Minneapolis, MN, USA

**Keywords:** Tutorial, clinical trials, SAS, STATA, R statistical software

## Abstract

**Background:**

Bayesian statistical approaches are extensively used in new statistical methods but have not been adopted at the same rate in clinical and translational (C&T) research. The goal of this paper is to accelerate the transition of new methods into practice by improving the C&T researcher’s ability to gain confidence in interpreting and implementing Bayesian analyses.

**Methods:**

We developed a Bayesian data analysis plan and implemented that plan for a two-arm clinical trial comparing the effectiveness of a new opioid in reducing time to discharge from the post-operative anesthesia unit and nerve block usage in surgery. Through this application, we offer a brief tutorial on Bayesian methods and exhibit how to apply four Bayesian statistical packages from STATA, SAS, and RStan to conduct linear and logistic regression analyses in clinical research.

**Results:**

The analysis results in our application were robust to statistical package and consistent across a wide range of prior distributions. STATA was the most approachable package for linear regression but was more limited in the models that could be fitted and easily summarized. SAS and R offered more straightforward documentation and data management for the posteriors. They also offered direct programming of the likelihood making them more easily extendable to complex problems.

**Conclusion:**

Bayesian analysis is now accessible to a broad range of data analysts and should be considered in more C&T research analyses. This will allow C&T research teams the ability to adopt and interpret Bayesian methodology in more complex problems where Bayesian approaches are often needed.

## Introduction

Statistical principles are widely used in study design and analysis, serving as a critical ingredient of the scientific method. Much of the statistical methodology used by clinical scientists, for example, standard errors, confidence intervals, tests of significance, multiple comparisons, and sample size estimation, has its roots in classical statistics [1]. However, in clinical and translational (C&T) research, the desire to integrate massive volumes and varieties of data types requires more complex statistical models. Estimation of these models is challenging and sometimes impossible using classical statistical approaches [2]. Advances in computing now allow development in a Bayesian framework, which offers a solution to estimation challenges. There are also compelling reasons to adopt a Bayesian framework for straightforward analyses (e.g., t-tests, standard linear/logistic regression). Reasons include an ability to integrate prior assumptions and historical knowledge about the model parameters using priors, a natural framework for incorporating measurement error (and misclassification) in covariates, and the fact that interpretation of posterior probabilities and credible intervals, the Bayesian analogs to p-values and confidence intervals, align with the definition many apply (incorrectly) to interpret p-values and confidence intervals [3]. Advances in a Bayesian framework specific to C&T research include adaptive designs [4–8], incorporating historical information [9], and more stable estimation properties in complex modeling [10–12].

Despite an increase in Bayesian approaches in methods development, adoption into C&T research has occurred more slowly. A Google Scholar search of publications in top statistics journals (Biometrics, Journal of the American Statistical Association, Biometrics, and Journal of the Royal Statistical Society: Series B) from 2010 to 2020 shows that the keyword Bayesian is linked with 30% of published articles over the same time period compared to top clinical journals (American Journal of Epidemiology, Journal of the American Medical Association, and New England Journal of Medicine) where the keyword Bayesian appears in 1.8% of the articles. This is perhaps because (1) interpretation of Bayesian analysis is not traditionally a competency of introductory statistics courses (a language gap) [13]; (2) Bayesian analyses involve the specification of prior distributions for the unknown parameters, adding a subjective element to analysis; and (3) Bayesian analysis implementation is perceived as more complex than classical approaches (a computation gap).

Progress to bridge the communication and training gaps has occurred [14–18]. This paper’s goal is to accelerate the transition of new methods into practice by improving the C&T researcher’s understanding and confidence in Bayesian analyses implementation. Our examples focus on regression analysis reflecting standard analyses (linear and logistic regression) used in C&T research. With increased understanding of a common analysis, we hope the conceptual learning is extendable to the more complex analyses in a Bayesian framework that occur in C&T research. We conduct an analysis using four commonly available statistical analysis packages/procedures using three software (R, SAS, STATA), which have packages making Bayesian regression achievable for those with some analytic expertise. In our example analyses, we investigate the impact of the prior including some accidental analytic mistakes commonly made but often not discussed in other introductory papers on the Bayesian approach. We also offer suggestions for evaluating whether a Bayesian regression analysis has been implemented properly.

## Methods

### Components of a Bayesian Analysis

In contrast to the classical statistical approach (a.k.a., frequentist) which conducts inferences using only the existing data, Bayesian analysis combines information in the data, through the likelihood, with prior information about the model parameters to obtain a combined assessment of uncertainty of these unknown quantities called the posterior (Table 1). Our examples are regression examples, and the parameters are the regression coefficients and the error variance for linear regression. Through an application of the well-known Bayes theorem, these components are linked as follows: P(parameters|data) ∝L (data|parameters) X P (parameters). The likelihood, L (data|parameters), and prior distribution of the parameters, P(parameters), are defined by the user. Computing the posterior distribution of the parameters, P (parameters|data), or distribution of the parameters given the data, is the analysis goal and analogous to estimating the regression coefficients and computing confidence intervals in frequentist analysis.

**Table 1.** Glossary of terms

Acceptance rate	The fraction of proposed samples from the sampler that is accepted
Autocorrelation	The sequential samples from the Markov chain are highly correlated with each other; this means the Markov chain is likely slowly traversing the sampling space because model parameters are highly correlated with one another
Autocorrelation plot	A plot showing pairwise correlation between MCMC iterations (*y*-axis) for different lags between iterations (*x*-axis); can be an indicator of poor sampling efficiency
Bayes theorem	A formal statistical method that includes conditional probabilities to quantify uncertainty of parameters of interest
Burn-in	A defined number of initial MCMC iterations discarded before creating diagnostic plots and summaries of the posterior distributions in order to minimize the effect on posterior inference
Chain	A series of random values from the range of the parameter’s distribution drawn by the MCMC sampler; in MCMC, it is common to call the simulation, or the sampling, a “chain” as shorthand because it is theoretically from a Markov chain
Convergence	The Markov Chain has reached the stationary (i.e., target) distribution
Credible interval (CrI)	The interval estimates for the parameter of interest with measurable probability (e.g., Equal-tailed or highest posterior density (HPD)); because Bayesian estimates are random, the credible interval can be interpreted as a probability range
Density plot	A histogram plot of the parameter’s posterior distribution
Error variance	Variance of the normally distributed error term in a linear regression model (also called residual error, residual variance)
Frequentist	Classical approach to statistical inference where the unknown parameters are held fixed
Informative prior	A prior distribution that may impact the posterior distribution relative to the likelihood; a prior that is not easily dominated by the likelihood function, e.g., Optimistic or skeptical priors determined by a subject-matter expert or previous literature
Likelihood	A statistical model that describes the distribution of the observed data and then used to update beliefs about the parameters when combined with the prior distributions
Markov Chain Monte Carlo (MCMC) algorithm	A set of algorithms for simulating, or randomly sampling, from a distribution even when the actual distribution cannot be mathematically derived
Mixing	(in relation to MCMC), describes the series, or chain, of random moves to explore the parameter’s range of values and relates to convergence of the MCMC
Non-informative prior	See *vague prior*
Posterior distribution	The distribution of the parameters conditioned on the trial data (i.e., observed data) and expresses the uncertainty in the parameters after observing the data; the updated beliefs about the parameters after the prior and the likelihood are combined using Bayes’ Theorem
Prior distribution	The current beliefs of a parameter summarized as the probability distribution
“Pseudo” vague prior	A prior that was initially thought to be non-informative but subsequently determined to substantially impact the posterior distribution, therefore, not truly vague
Sampler (or sampling algorithm)	An algorithm or sampling method employed to obtain random samples from the target distribution; see *Markov Chain Monte Carlo (MCMC) algorithm*
Starting value	The initial value for the MCMC sampler for beginning the series of sampling draws; the value can be a mean and a variance
Trace plot	A plot which has the value of the parameter on the *y*-axis at each MCMC iteration (*x*-axis); an ideal plot will show convergence where the parameter is oscillating around the mode of the distribution
Vague prior	A prior distribution that will have minimal impact on the posterior distribution relative to the likelihood function, e.g., a flat distribution relative to the likelihood
Wandering	See *mixing*

**Likelihood**: The likelihood is related to the model, or distribution, of the data (i.e., outcome) in the study, which is the case in both classical and Bayesian approaches. In linear regression, the standard model of the data (or outcome) for participant *i* is assumed to be a normal distribution with a mean equal to *β*_0_ + *β*_1_ *X*_1*i*_ + ⋯ + *β*_*k*_ *X*_*ki*_, where the *X*_*ji*_’s are the variables of interest measured on participant *i*, and a variance of *σ*_*e*_^2^. Given the independence assumption, the model for all participants is the product of these normal distributions. The likelihood function is the same mathematical function except now the data are fixed at the observed data and the function is studied as a function of the parameters. Thus, the likelihood function characterizes how “likely” the parameters are for a given set of data. In logistic regression, the likelihood is based on a binomial distribution where a logit transformation of the probability of success (*p*) is linked to variables of interest as follows: 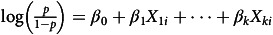 . By selecting a linear or logistic regression analysis, the likelihood is typically specified by the package/procedure. The analyst selects the variables of interest when specifying the model in the statistical code.

**Figure 1.**
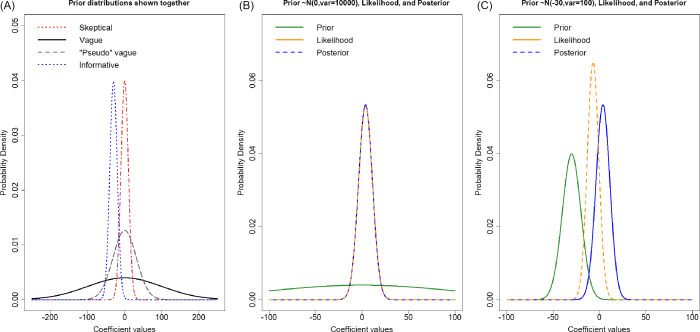
Panel A). The four priors on the treatment effect in the linear regression. The black solid line is the vague prior and shows an even weight for the largest range of the values. The gray dashed line is the “pseudo” vague prior, which has more weight around zero over a fairly large range of treatment effect values (but does not cover the range of values consistent with distribution of the outcome and thus, informative for the intercept). The red medium dashed line is the skeptical prior with much more weight centered around small treatment effects and the blue dotted line is the optimistic prior with nearly all its weight on treatment effects less than zero. Panels B and C). The linear regression coefficient values of the treatment group of the prior (green solid line), likelihood (solid blue), and posterior (orange dashed). Panel B plots values from the vague prior scenario showing that this prior specification does not pull the coefficient from the likelihood, as the two density curves are nearly identical. Panel C plots the informative prior scenario showing that an informative prior can influence the posterior from the likelihood, and the posterior is a combination of the prior and likelihood curves.

**Prior Distribution on Parameters**: Prior distributions on the parameters quantify a belief in the parameter values before observing any study data, with the spread of the distribution quantifying the strength of those beliefs. In linear and logistic regression, the main parameters are the beta coefficients and typical distributions for the prior are normal distributions. Normal distributions are chosen for mathematical convenience but also allow for a full range of the parameters (−∞,+∞) to be accommodated in the prior. In linear regression, there is a prior distribution chosen for the inverse of the model error variance (model precision), which is typically a gamma distribution to take on only non-negative values. Other choices include truncated normal, uniform, half T, or half Cauchy. There is vast literature on defining prior distributions and eliciting prior distributions that capture a scientist’s beliefs about parameter values [19–21]. In practice, we recommend C&T teams discuss different forms of prior knowledge, including a clinician’s perspective from past research or clinical care and information published from other studies. We also recommend analysis with several different priors identified with this approach to formally understand how conclusions may change based on a range of a priori assumptions C&T researchers may have about parameters. In this work, we specifically investigate various types, or “strengths,” of priors (Fig. 1). These ranged from vague with a wide, flatter distribution to skeptically and optimistically informative depending on the value of the mean in the priors for the betas. Priors with means set equal to 0 with more prior probability of no treatment difference (created by a smaller or tighter variance) compared to a vague prior have been labeled skeptical and quantify the strength of a clinician’s prior belief that the treatment will not have an effect. Priors with means not equal to 0 (negative or positive depend on clinical context) with more prior probability of a treatment difference (again, created with a smaller variance) compared to a vague prior have been labeled optimistic toward associations and quantify the strength of a clinician’s prior belief that the treatment will have an effect. As discussed earlier, the value of the mean can be chosen based on clinician input, historical data (e.g., previous trials), or biological plausibility. The labeling of priors as vague, skeptical, and optimistic depends directly on the scale of the outcome. For example, in one problem a variance of 1000-units^2^ could be quite large and vague while another outcome with large values and highly variable measurements may need a much larger variance to be vague.

**Posterior distribution**: The posterior distribution is the distribution of the parameters conditioned on the data and expresses the uncertainty in the parameters after observing the data. As noted earlier, the posterior is proportional to the likelihood times the prior emphasizing that, on a log scale, the information about the parameters in the posterior is essentially the sum of information in the prior and the data. It is common to summarize the posterior by its mean, median, or mode and credible intervals. For example, a 95% equal-tailed probability or credible interval is computed by finding the 2.5^th^ and 97.5^th^ percentiles of the posterior distribution. This interval is interpreted as the 95% posterior probability that the parameter is between the bounds given the observed data. We note that this interpretation is commonly misapplied to the classical confidence interval (i.e., if we repeated sampling infinitely, the resulting 95% confidence intervals would contain the true population value 95% of the time; therefore, the correct interpretation is we are 95% confident that the bounds contain the parameter value) [22–25]. Another commonly used credible interval is the highest posterior density (HPD) interval, which is the interval with the smallest interval width among all credible intervals. Using the posterior distribution, one can also compute posterior probabilities of ranges of parameter values aligning with clinically meaningful differences between the treatment arms. An example might be the posterior probability that the treatment has a higher (or lower) mean compared to the control group.

### Computing the Posterior

In Bayesian regression analysis, the posterior is rarely a known distribution. Instead, Markov Chain Monte Carlo (MCMC) algorithms are used to simulate samples from the posterior. MCMC is a set of algorithms for sampling from a distribution even when the actual distribution cannot be mathematically derived [26–28]. Gibbs sampling and Metropolis-Hastings (M-H) are two common algorithms among many other approaches [29–31]. Detailed descriptions are available elsewhere [28]. The beauty of software development in the past decade is that, in SAS, *R*, or STATA, the user only needs to specify the variables in the regression model, similar to classical analysis, and distribution of the priors. The programs assemble the math for the MCMC algorithms behind the scenes for the user making Bayesian implementation highly feasible for most analysts. Code for each package and software are publicly available at https://github.com/nichole-carlson/BayesianClinicalResearch.

The output of MCMC algorithms is a rectangular dataset where columns are a sample from the posterior distribution of a model parameter and each row is a single iteration from the algorithm. The set of samples is often called “chains” reflecting that the samples are derived from a Markov chain, the mathematical system behind MCMC. By design, chain iterations are serially correlated (called autocorrelation). Assessing the autocorrelation strength is useful for investigating algorithm performance including likely convergence to the posterior and full posterior exploration. In practice, some run the MCMC algorithm 2–3 times, each a unique chain of sampling with different random starting values to further assess convergence. However, once adequate algorithm performance is determined, a single longer chain is used for final posterior estimation [32].

**Figure 2.**
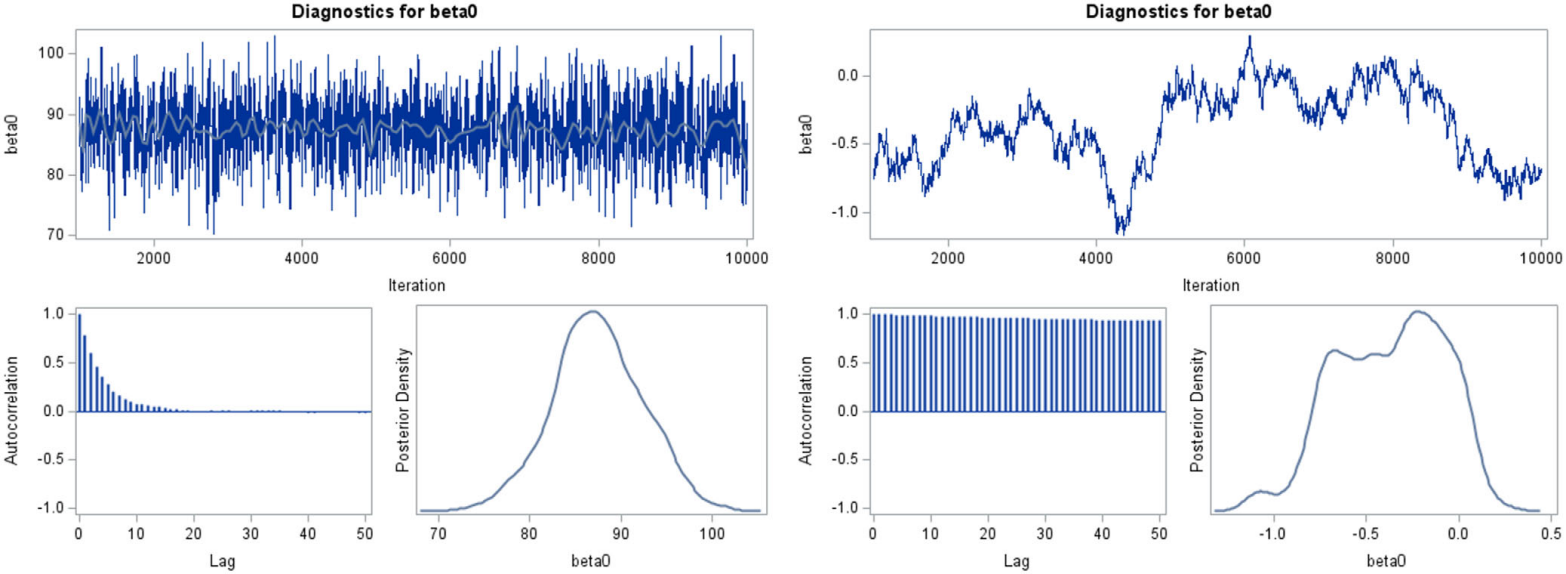
Diagnostic plots for various scenarios. The left panel indicates convergence is likely and the right where convergence is less likely and the MCMC algorithm is modified. The top figure in each panel is a trace plot. The bottom-left figure is an autocorrelation plot, and the bottom-right figure is a posterior density plot. These were generated by SAS PROC MCMC. Similar graphics are available for the other software.

To investigate these concepts, there are several graphical diagnostics to be assessed for each column. The most common diagnostic plots are trace, autocorrelation, and density plots (Fig. 2). A trace plot has parameter values on the *y*-axis for each iteration (*x*-axis). The autocorrelation plot is the pairwise correlation (*y*-axis) between parameter values for different lags between iterations (*x*-axis). When multiple chains are run, we first assess whether, after a suitable number of iterations, the trace plots start to overlap. Lack of overlap raises the possibility that convergence has not been achieved in the number of iterations selected. Assuming the trace plots eventually converge, further graphical assessment (and posterior summary measures) should not be visually influenced by a particular starting value. Thus, a portion of the initial algorithm iterations (called burn-in) is removed. The burn-in is often ∼ 10% of the iterations with no firm recommendation. The trace plot looks like random noise when convergence is likely (Fig. 2, top-left panel) versus having strong patterns or long stretches of wandering (Fig. 2, top-right panel). The autocorrelation plot exhibits a steep decline as the lags increase (Fig. 2, bottom-left, both panels). Strong patterns in the trace plots and long lags with a high correlation indicate poor mixing and a lower possibility of convergence or limited exploration of the posterior. The results may not be valid. Density (or histogram) plots of the parameters should have a smooth shape with a single mode (Fig. 2, bottom-right, both panels). In typical regression analyses, other bimodal or unusual patterns may also indicate lack of convergence. There are other diagnostics, e.g., the Gelman-Rubin statistic, for assessing convergence [33,34]. For simplicity, we focused on visual diagnostics.

After verifying the simulation has likely converged to the posterior distribution, the posteriors are summarized using the mean and measures of variability described by credible intervals and/or posterior standard errors and standard deviations.

### Application of Bayesian Analysis to a Clinical Trial

Here we draft a formal methods section for our illustrative application as an example for the reader to follow when writing their own sections in clinical journals. Key components to consider are in Table 2. Our anesthesiologist clinical research collaborators were interested in evaluating how sublingual sufentanil, a novel opioid medication for moderate to severe pain, performed relative to the existing standard of care therapy of intravenous (IV) fentanyl. The study details are published elsewhere [35]. In brief, 75 patients were randomized to two study arms, and 66 were included in the per-protocol illustrative analysis. The exposure of interest was drug treatment with either sublingual sufentanil or IV fentanyl (the referent group). The primary continuous outcome was time to readiness for discharge after arrival in post-anesthesia care unit (PACU; in total minutes), and the primary dichotomous outcome was if a preoperative nerve block was administered (yes or no; probability of yes was modeled). All modeling was performed in parallel with R v4.2.1 (Vienna, Austria), SAS version 9.4 (Cary, NC, USA), and STATA version 17.0 (College Station, TX, USA).

**Table 2.** Statistical components to include in a Bayesian data analysis plan

a. The outcome, primary variable of interest, and covariates with labels as to their function as adjustment for confounding, precision variables, mediators, etc.
b. The regression modeling framework being used (linear regression, logistic regression, or other).
c. Prior distribution with parameters; a description of non-informative to informative and a justification for the selection when appropriate; multiple priors should be investigated over a range of assumptions regarding a priori beliefs about the values of the parameters.
d. Package and software version with specifics about the MCMC algorithm used.
e. Chain/iteration length and number and burn-in.
f. Parameter initialization values.
g. Assessment of convergence.
h. A description of how posteriors will be summarized.

### Analysis of Time to Readiness for Discharge

Linear regression was used to model the association between treatment and discharge with and without adjustment for covariates. In the unadjusted models, the intercept represented the mean time to readiness for discharge in the IV fentanyl treatment group and the treatment coefficient represented the differences in the mean time to readiness for discharge from PACU between the sufentanil and IV fentanyl treatment groups. The adjusted models included centered procedure length (minutes) and sex (dichotomous).

The primary prior chosen for our analysis was a vague N(mean = 0, variance = 10,000) prior giving almost equal a priori weight to a large range of plausible values (Table 3; Fig. 1 panel A). In this prior distribution, there was an 80% a priori probability that the treatment difference was between −128 and 128 minutes (the values used to compute this range are the 10^th^ and 90^th^ percentiles of the prior distribution) and 50% of the a priori treatment effect values were less than 0. This same prior was also chosen for the regression coefficients on procedure length and sex in the adjusted model. The vague priors were considered conservative and similar to traditional analytic approaches.

**Table 3.** Specified priors

	Vague	Skeptical	Optimistic	“Pseudo” Vague
Linear regression				
Intercept	*N* (0, 10,000)	*N* (0, 10,000)	*N* (0, 10,000)	*N* (0, 1000)
Drug group	*N* (0, 10,000)	*N* (0, 100)	*N* (−30, 100)	*N* (0, 1000)
Covariates	*N* (0, 10,000)	*N* (0, 10,000)	*N* (0, 10,000)	*N* (0, 1000)
Sigma[2]	IG (0.01, 0.01)	IG (0.01, 0.01)	IG (0.01, 0.01)	IG (0.01, 0.01)
Logistic regression				
Intercept	*N* (0, 10)	*N* (0, 10)	*N* (0, 10)	*N* (0, 1)
Drug group	*N* (0, 10)	*N* (0, 2)	*N* (−0.5, 2)	*N* (0, 1)
Covariates	*N* (0, 10)	*N* (0, 10)	*N* (0, 10)	*N* (0, 1)

IG = inverse gamma (shape, scale); N = normal (mean, variance).

We also considered informative optimistic and skeptical priors (Table 3; Fig. 1 panel A) reflecting two common prior beliefs held by the clinical investigators. The optimistic prior was a *N* (−30, 100) reflecting a greater a priori belief in a clinically meaningful treatment difference. In this setting, the treatment difference (−30 minutes) and variance were selected based on the assumptions of the a priori power calculation, which relied upon historical data and clinician input. The optimistic prior had an 80% a priori probability of a treatment difference between −42.8 and −17.2 minutes with 99.8% of the a priori treatment differences less than 0. The skeptical prior was a *N* (0, 100) reflecting a greater a priori belief in no treatment effect with the variation about zero chosen from the a priori power calculation. The skeptical prior had an 80% a priori probability of a treatment difference between −12.8 and 12.8 minutes. In both scenarios, vague priors of *N* (0, 10000) were chosen for the regression coefficients on the covariates and intercept in the adjusted model.

We also considered a *N* (0, 1000) prior for all the parameters in the regression model (not just the treatment coefficient), representing the same numerical values but specified on different units. We classified this as a “pseudo” vague prior in that it was intended to be vague for all parameters, but further investigation showed it was informative for the intercept term. We selected this prior because it is a common choice among early adopters of Bayesian analyses, often thought to be a vague prior without careful consideration of the units of the outcome. Upon visual inspection of the range of parameter values allowed for the intercept compared to the distribution of the outcome, this prior was not vague and quite informative. This represents a case scenario that does not perform as intended with unintended high bias in the treatment coefficient and an example of what can go wrong in naive implementations.

For all scenarios, the model error variance had an inverse-gamma (IG) prior with a shape = 0.01 and a scale = 0.01, which are common choices for a vague prior.

PROC MCMC and PROC GENMOD with a BAYES statement were used to sample the posterior in SAS. We employed the default MCMC algorithms within each software, including N-Metropolis (SAS, PROC MCMC), Gibbs for linear regression (SAS, GENMOD + BAYES), random walk Metropolis-Hastings (STATA), and No-U-Turn sampling (RStan, BRMS package).

To improve comparability across results, parameters were initialized with regression coefficients set to “0” and the model error variance set to “1.” In practice, software default settings for initialization values are sufficient. RStan and STATA ran two MCMC chains, while SAS used a single chain. Each chain was run for 10,000 iterations including 1000 iterations discarded as burn-in and all values were stored. Software default settings were used for the target acceptance rate in Metropolis-Hastings algorithms. Convergence was assessed visually using trace, autocorrelation, and histogram plots of the posterior distributions for each parameter. Results were summarized using posterior means and 95% HPD credible intervals (CrI). We also computed the posterior probability of a positive treatment difference represented by a reduction in the mean time to readiness for discharge from PACU (i.e., treatment difference < 0).

We repeated linear modeling in a classical framework in each software. Results were presented as estimates and 95% confidence intervals.

### Analysis of Administration of Preoperative Nerve Block

Logistic regression was used to model the association between treatment and the odds of administering preoperative nerve block. Unadjusted and adjusted models were specified.

As above, we considered several priors on the treatment effect. The primary prior was a vague *N* (0, 10) with 80% prior probability of an odds ratio (OR) between 0.02 and 60. Our optimistic prior was a *N* (−0.5, 2) with an 80% prior probability of an OR between 0.1 and 3.7 and a 64% prior probability of an OR less than 1. Our skeptical prior was a *N* (0, 2) with an 80% prior probability of an OR between 0.2 and 6.0 and a 49% prior probability of an OR less than 1. We used vague *N* (0, 10) for the covariate regression coefficients in the adjusted models. The “pseudo” vague prior was a *N* (0, 1) for the treatment and covariate regression coefficients. The MCMC algorithms were as above except for SAS GENMOD + BAYES which used a Gamerman algorithm. The posteriors were summarized using means and 95% HPD credible intervals and exponentiated to ORs for a standard clinical interpretation. The posterior probability of a reduced odds of nerve block usage (i.e., OR < 1) was also computed. Analyses were repeated in the classical framework in each software. Results were presented as OR with 95% CIs.

## Results

### Primary Analysis Using a Vague Prior, SAS PROC MCMC

Here we provide a summary of the results written suitably for a clinical publication to provide an examples of writing Bayesian results. We hope the reader finds reassurance that much of the text is like that of a classical approach.

Supplemental Table S1 shows the demographics of the study population by treatment group. Representative diagnostic plots can be found in supplementary material (Figures S1–S12). No convergence issues were observed. Those in the sublingual sufentanil group had on average a 3.8 minute longer time to readiness for discharge compared to the fentanyl group (results presented from SAS PROC MCMC: 95% HPD CrI: −11.1, 18.2). The 95% HDP CrI is interpreted as the difference between the two treatment groups having a 95% chance of falling between a decrease of 11.1 minutes and an increase of 18.2 minutes. The posterior probability of a decrease in time to readiness for discharge was 29.7% indicating there is not a high posterior probability that the new drug reduces the outcome. Results were consistent after adjustment (Posterior Mean = 6.7 min; 95% HDP CrI: −8.0, 22.7; posterior probability = 22.3%).

In addition, sublingual sufentanil reduced the odds of preoperative nerve block by 39% on average (Posterior Mean OR: 0.61, 95% HPD CrI: 0.08, 1.32). The posterior probability that sublingual sufentanil reduced the odds of preoperative nerve block was 87.5%, a high posterior probability that the new drug reduces the odds of the outcome. The results were only slightly attenuated after adjustment (Posterior Mean OR: 0.74; 95% HPD CrI: 0.09, 1.75; posterior probability = 77.9%).

### Comparison of Results Between Software Programs

**Figure 3.**
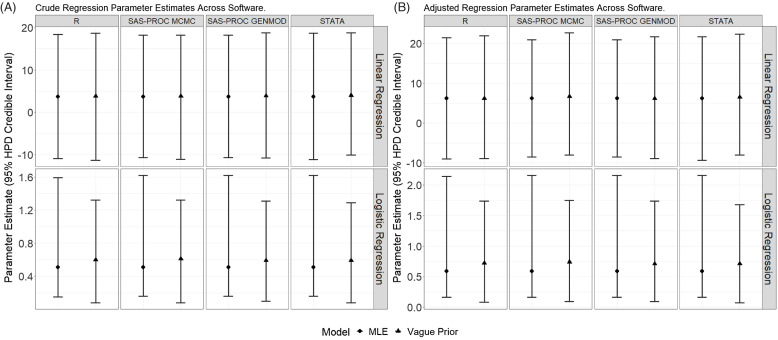
A comparison of crude (panel A) and adjusted (panel B) treatment effects across different software programs. Circle is the MLE and 95% confidence interval. Triangle is the posterior mean and 95% HDP CrI. Logistic regression results are odds ratios. Linear regression vague prior ∼N (0, 10,000); logistic regression vague prior ∼N (0, 10); MLE = maximum likelihood estimate.

Fig. 3 presents the posterior means and 95% HPD CrIs for the linear and logistic regression analyses for the treatment variable from the unadjusted and adjusted analyses with each software package for vague prior and the classical analysis. Reassuringly, the findings were similar regardless of software. This pattern is consistent for the other priors and the adjusted analyses (see supplemental materials) except for the “pseudo” vague prior where RStan was less influenced by the prior on the intercept compared to the other algorithms.

### Comparison of Results Across Different Priors

**Figure 4.**
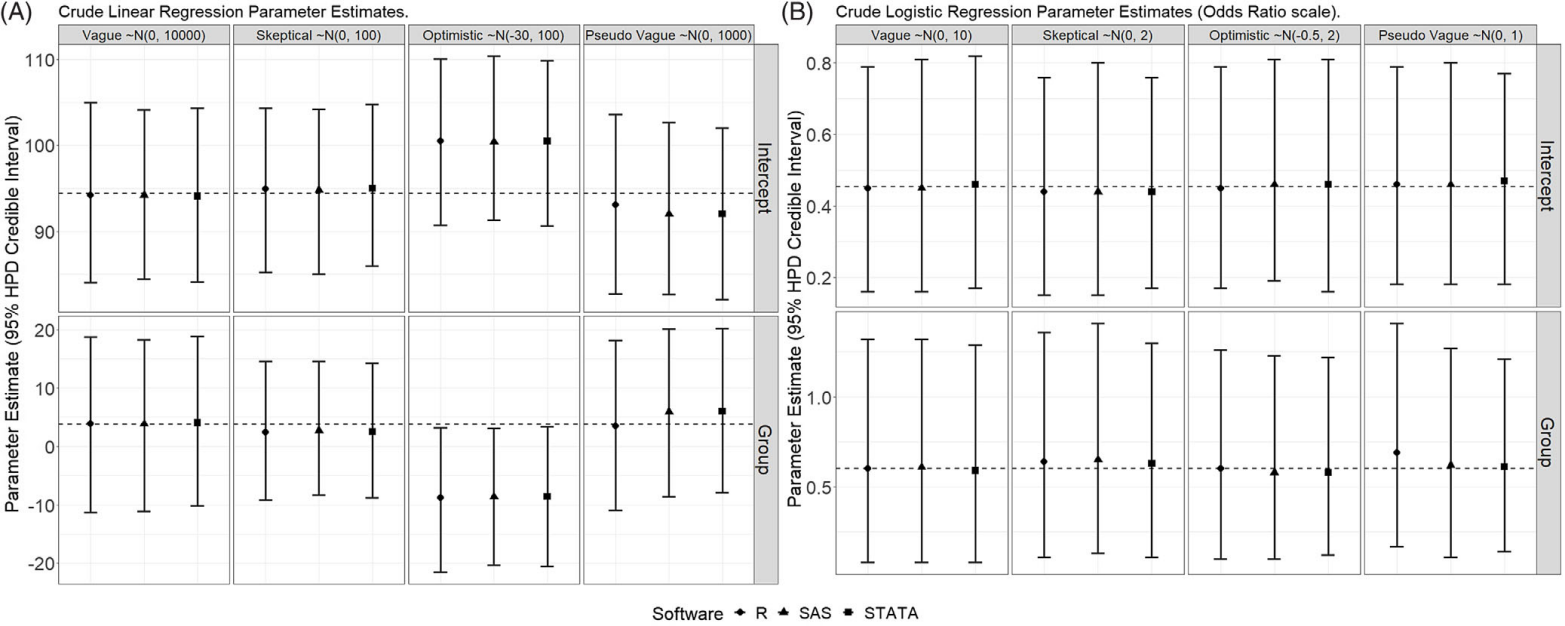
A comparison of posterior mean and 95% credible intervals (CrI) for the three software programs (R, SAS PROC MCMC, and STATA) for the crude linear regression (panel A) and crude logistic regression (panel B). Dashed reference lines reflect the parameter estimates generated from MLE frequentist models. The intercept parameter was specified with priors of ∼N (0, 10,000) [linear regression] and ∼N (0, 10) [logistic regression] within both skeptical and informative scenarios; otherwise, the intercept prior was specified the same as the main effect.

Fig. 4 presents the posterior means and 95% HDP CrIs for the simple linear and logistic regression analyses for the intercept and treatment variable from the unadjusted analyses. The posterior means for the intercept and treatment effect were very similar for the vague and skeptical priors. We note that the posterior means for the intercept and treatment effect were nearly identical for the vague priors and the MLE. This highlights how, with a suitably vague prior, the data (through the likelihood) are allowed to dominate the estimation (Fig. 1, panel B). For the unadjusted linear regression with the “pseudo” vague prior, the intercept mean was pulled toward the prior mean [Posterior Mean = 92.0; 95% HPD CrI: 82.6, 102.7] and the treatment effect biased higher compared to the truly non-informative priors [Posterior Mean = 5.9; 95% HPD CrI: −8.6, 20.1]. Even though a variance of 1000 seemed large and covered a wide range of parameter values, it was informative for the intercept, which was far from 0 and not given an equal weight as smaller values in the prior (i.e., an accidentally non-sensical prior for an intercept). Given the sample size of the trial was modest, the informative optimistic prior estimated a treatment effect that was larger than the non-informative priors but smaller than the mean of the prior (Fig. 1, panel C). This reflects how the data reweight information in the prior to arrive at posterior estimates. It also indicates that this trial was inconsistent with the a priori assumption about the treatment effect.

## Discussion

The ability of C&T researchers to understand statistical concepts to advance medical discovery and to use data driven-decisions in practice is well recognized, as evidenced by statistical curricula in medical school and continuing education programs. However, innovation occurs at a far faster pace than adoption into practice. The advancement of new approaches for analyzing data also develops at a faster pace than graduate curriculum or practice allows. Publications targeted to subject areas of research are another venue to increase the adoption of innovation into practice. Our goal was to help C&T researchers adopt newer statistical methods in practice, especially those developed using a Bayesian framework.

We focused on introducing C&T researchers to the key elements of Bayesian linear and logistic regression analyses including specifying the model and the prior distributions of the parameters and computing the posterior distribution of the parameters. We also provided a high-level overview of the computational engine behind Bayesian analysis and MCMC algorithms. We offered guidelines on analysis plan components and provided a straightforward example of a clinical trial’s results section, which has not been highlighted as much in other papers. We hope this practical guide gives C&T researchers an ability to evaluate the Bayesian statistical analysis plans developed by their biostatisticians or other data analysis-focused team members. We hope it also allows C&T researchers to confidently and critically evaluate Bayesian analyses in the literature and encourage others to include all the details necessary to evaluate, reproduce, and interpret Bayesian analyses.

We chose to focus the application on a traditional two-arm clinical trial with both continuous and binary outcomes even though results with vague priors do not differ from classical approaches. This was done to develop an understanding of Bayesian approaches in common settings, which we hope will translate to confidence in adopting Bayesian approaches in complex settings where traditional/classical estimation approaches may have significant limitations or be impossible. Examples include high dimensional problems [36], variable selection [37,38], clustering [39], and incorporating historical data in clinical trials [40].

We found STATA the most approachable for those without programing experience owing to its user-friendly point-and-click interface and accessible documentation for common Bayesian analysis needs. However, determining how to reproducibly generate posterior summaries and manipulate the posterior means, such as variable transformation, was not straightforward. Additionally, calculating the posterior probabilities on the non-transformed [log(Odds)] versus transformed (OR) scale generated different results which is not intuitive. SAS was straightforward with the best documentation for simple and complex applications of Bayesian commands and has flexibility between two different PROCs; although it requires programing knowledge. PROC MCMC was preferred due to explicit options for setting chain initial values and prior specifications compared to PROC GENMOD + BAYES. However, SAS does not offer an easy approach (i.e., a single argument) for running multiple chains, unlike R and STATA, so the analyst must create a macro program to execute and combine multiple chains to conduct analysis and diagnostics. RStan was also simple but required programing knowledge, and its documentation seemed less complete or challenging to interpret in some instances. Further, compared to STATA and SAS (and without optimization), compiling, and summarizing the posterior took longer which should be considered if working with “big data.”

Implementing the Bayesian approach has challenges. It was difficult identifying the MCMC algorithm details and understanding how the parameters were initialized and the default parameters in priors. This could lead to a perception that these details are not important in practice. A specific example was the parameters in the prior for the model error between PROC MCMC and PROC GENMOD. Both have optional statements to set these values and can fit models without explicit specification of this parameter. However, there is a default model error parameter in PROC GENMOD, while PROC MCMC can run without specification and the parameter is not displayed in the output when excluded from the code’s parameter list. This makes it easy to misspecify or accidentally exclude its prior specification. These differences in prior specification resulted in a range of MCMC performance and slight differences in posterior estimates. We strongly encourage C&T researchers to set the parameter values in all priors.

We also were reminded how in regression analyses it is easy to be informative for the intercept if one is not careful about investigating the scale of the outcome and range of the prior. If the user a priori wants to have non-informative priors, which would be common for the intercept, we recommend that the analyst compute the mean of the outcome for continuous measures and plot the prior distribution for the intercept to confirm that it is non-informative over the appropriate range.

Simulation-based approaches start with a random seed for simulating from the distributions. Approaches for setting seeds vary and even in cases where the same seed is set, different random generators are used across software to simulate the same distributions. Thus, reproducibility between software is often not possible. To achieve reproducibility within a package, we recommend setting the seed to create a known and fixed number. Each software had an optional explicit argument to set a seed value within the main syntax for a Bayesian framework.

One major concern with Bayesian analysis is sensitivity to the priors. We believe this is a benefit because the prior allows us to transparently specify our prior beliefs about the parameters. The approach used in this analysis was based on clinical knowledge obtained from the clinical investigators’ power calculations. There are other approaches to specifying informative priors including clinical practice guidelines, clinician expertise [20], a sensitivity analysis including both skeptical and conservative priors [41,42], and theory based on how much the prior and data are balanced in the posterior estimate [19,43]. We strongly encourage C&T researchers to use their domain knowledge to construct a range of priors incorporating multiple values representing skeptical to optimistic opinions and include all results in the write-up. We also note the trial investigated in this paper was a modest-sized trial allowing us to highlight the effect priors can have on the posterior. As sample size increases, we expect differences in the posterior across priors to become less pronounced. This reflects increased weighting of the likelihood versus the prior in the mathematics computing the posterior.

There are many Bayesian concepts not covered including model selection using the deviance information criterion (i.e., DIC, the Bayesian analog to AIC in classical methodology), Bayes factors, adaptive MCMC, and model diagnostics using posterior predictive distributions. Readers desiring more knowledge are referred to more extensive texts [19,41], and SAS help manuals [44].

In summary, statistical software now allows Bayesian analyses as a standard approach. We have provided an overview of regression analysis components, and shown how to interpret Bayesian analyses, and results are quite stable for a wide range of prior distributions. Informative priors are useful for incorporating existing clinical or prior trial knowledge, knowledge translation, or existing scientific knowledge such as biologically plausible ranges of values. These cases are of high interest in translational science initiatives, such at the National Center for Advancing Translational Science in the National Institutes of Health, which is interested in knowledge translation from animal to human studies [45], and at the Food and Drug Administration [46].

## Supporting information

Gunn-Sandell et al. supplementary material Gunn-Sandell et al. supplementary material

## Supplementary material

The supplementary material for this article can be found at https://doi.org/10.1017/cts.2023.689.
