# Supplementary material for: A practical guide to adopting Bayesian analyses in clinical research
Source: J Clin Transl Sci. 2023 Dec 7;8(1):e3. doi: 10.1017/cts.2023.689 (PMC10877520; doi:10.1017/cts.2023.689)

**SUPPLEMENTAL MATERIAL**

**“A Practical Guide to Adopting Bayesian Regression Analyses in Clinical Research”**

Supplemental Table S1. Trial population characteristics.

|  | **Drug Group** | | **Total,**  **n=66** |
| --- | --- | --- | --- |
| **Factor** | **Sublingual Sufentanil,**  **n=33** | **Fentanyl,**  **n=33** |  |
| **Sex, Female**, *N (%)* | 20 (60.6) | 17 (51.5) | 37 (56.1) |
| **Preoperative nerve block, Yes**, *N (%)* | 6 (18.2) | 10 (30.3) | 16 (24.2) |
| **Time to readiness for discharge after arrival in PACU**, *mean (SD)* | 98.1 (31.7) | 94.4 (29.0) | 96.2 (30.2) |
| **Procedure length (minutes)**, *mean (SD)* | 57.0 (41.6) | 84.6 (54.3) | 70.8 (50.0) |

Table S2: Influence of priors on unadjusted linear regression results for each package and software not presented in the main results

|  | MLE  (non-Bayesian) | Vague  N(0, 10000) | Skeptical  N(0, 100) | Optimistic  N(-30, 100) | Pseudo-Vague  N(0, 1000) |
| --- | --- | --- | --- | --- | --- |
|  | **Beta Coefficient (95% CI)** | **Posterior Mean Beta Coefficient (95% HPD Credible Interval)** | | | |
|  | SAS - PROC MCMC | | | | |
| Intercept | 94.4 (84.2, 104.6) | 94.2 (84.4, 104.1) | 94.8 (85.0, 104.2) | 100.4 (91.3, 110.4) | 92.0 (82.6, 102.7) |
| Drug group (Fentanyl=Ref) | 3.7 (-10.7, 18.2) | 3.8 (-11.1, 18.2) | 2.7 (-8.3, 14.5) | -8.6 (-20.3, 3.1) | 5.9 (-8.6, 20.1) |
|  | STATA | | | | |
| Intercept | 94.4 (83.8, 104.9) | 94.1 (84.1, 104.3) | 95.0 (85.9, 104.8) | 100.5 (90.6, 109.9) | 92.0 (82.0, 102.0) |
| Drug group (Fentanyl=Ref) | 3.7 (-11.2, 18.7) | 4.0 (-10.1, 18.8) | 2.5 (-8.8, 14.2) | -8.6 (-20.5, 3.4) | 6.0 (-7.9, 20.2) |
|  | RStan | | | | |
| Intercept | 94.4 (84.0, 104.7) | 94.2 (84.0, 105.0) | 94.9 (85.2, 104.3) | 100.5 (90.7, 110.1) | 93.1 (82.7, 103.6) |
| Drug group (Fentanyl=Ref) | 3.7 (-10.9, 18.4) | 3.8 (-11.3, 18.7) | 2.4 (-9.2, 14.5) | -8.8 (-21.5, 3.2) | 3.5 (-10.9, 18.1) |

Table S3: Influence of priors on adjusted linear regression results for each package and software not presented in the main results

|  | MLE  (non-Bayesian) | Vague  N(0, 10000) | Skeptical  N(0, 100) | Optimistic  N(-30, 100) | Pseudo-Vague N(0, 1000) |
| --- | --- | --- | --- | --- | --- |
|  | **Beta Coefficient (95% CI)** | **Posterior Mean Beta Coefficient (95% HPD Credible Interval)** | | | |
|  | SAS - PROC MCMC | | | | |
| Intercept | 89.1 (76.3, 101.9) | 88.2 (73.8, 101.2) | 90.3 (76.4, 102.6) | 95.7 (82.4, 107.9) | 85.6 (73.1, 98.3) |
| Drug group (Fentanyl=Ref) | 6.2 (-8.5, 20.9) | 6.7 (-8.0, 22.7) | 3.9 (-7.8, 17.4) | -7.7 (-20.8, 4.3) | 8.5 (-6.3 23.4) |
| Sex  (Male = Ref) | 7.2 (-7.2, 21.7) | 8.1 (-8.7, 23.8) | 6.7 (-8.5, 22.8) | 8.0 (-6.6, 23.6) | 9.2 (-4.1, 24.1) |
| Procedure Length | 0.11 (-0.04, 0.3) | 0.12 (-0.02, 0.3) | 0.10 (-0.05, 0.3) | 0.07 (-0.09, 0.2) | 0.12 (-0.02, 0.3) |
|  | STATA | | | | |
| Intercept | 89.1 (75.6, 102.5) | 88.6 (76.0, 102.5) | 89.9 (77.4, 102.2) | 95.8 (82.7, 108.0) | 85.2 (71.2, 98.5) |
| Drug group (Fentanyl=Ref) | 6.2 (-9.3, 21.7) | 6.5 (-8.0, 22.3) | 4.2 (-9.0, 15.5) | -7.8 (-20.1, 5.3) | 8.4 (-6.0, 24.0) |
| Sex  (Male = Ref) | 7.2 (-8.0, 22.4) | 7.4 (-6.3, 22.0) | 7.6 (-8.0, 22.1) | 8.0 (-5.9, 24.0) | 9.6 (-4.5, 24.1) |
| Procedure Length | 0.11 (-0.04, 0.3) | 0.12 (-0.03, 0.3) | 0.11 (-0.04, 0.3) | 0.07 (-0.09, 0.2) | 0.12 (-0.03, 0.3) |
|  | RSTAN | | | | |
| Intercept | 89.1 (75.9, 102.3) | 88.9 (75.9, 102.6) | 90.0 (77.6, 103.0) | 95.6 (82.4, 108.4) | 88.1 (75.0, 101.6) |
| Drug group (Fentanyl=Ref) | 6.2 (-9.0, 21.4) | 6.2 (-8.9, 21.9) | 3.9 (-7.8, 16.2) | -8.0 (-20.8, 4.7) | 5.9 (-9.3, 20.7) |
| Sex  (Male = Ref) | 7.2 (-7.7, 22.1) | 7.2 (-7.8, 21.9) | 7.4 (-7.7, 22.8) | 7.9 (-7.8, 22.9) | 6.8 (-8.1, 21.5) |
| Procedure Length | 0.11 (-0.04, 0.3) | 0.11 (-0.04, 0.3) | 0.11 (-0.05, 0.3) | 0.07 (-0.09, 0.2) | 0.11 (-0.04, 0.3) |

Table S4: Influence of priors on unadjusted logistic regression results for each package and software not presented in the main results

|  | MLE  (non-Bayesian) | Vague  N(0, 10000) | Skeptical  N(0, 100) | Optimistic  N(-30, 100) | Pseudo-Vague  N(0, 1000) |
| --- | --- | --- | --- | --- | --- |
|  | **OR (95% CI)** | **Posterior Mean OR (95% HPD Credible Interval)^b^** | | | |
|  | SAS - PROC MCMC | | | | |
| Intercept | 0.43 (0.21, 0.91) | 0.45 (0.16, 0.81) | 0.44 (0.15, 0.80) | 0.46 (0.19, 0.81) | 0.46 (0.18, 0.80) |
| Drug group (Fentanyl=Ref) | 0.51 (0.16, 1.62) | 0.61 (0.08, 1.32) | 0.65 (0.13, 1.41) | 0.58 (0.10, 1.23) | 0.62 (0.11, 1.27) |
|  | STATA | | | | |
| Intercept | 0.43 (0.21, 0.91) | 0.46 (0.17, 0.82) | 0.44 (0.17, 0.76) | 0.46 (0.16, 0.81) | 0.47 (0.18, 0.77) |
| Drug group (Fentanyl=Ref) | 0.51 (0.16, 1.62) | 0.59 (0.08, 1.29) | 0.63 (0.11, 1.30) | 0.58 (0.12, 1.22) | 0.61 (0.14, 1.21) |
|  | RStan | | | | |
| Intercept | 0.43 (0.20, 0.89) | 0.45 (0.16, 0.79) | 0.44 (0.15, 0.76) | 0.45 (0.17, 0.79) | 0.46 (0.18, 0.79) |
| Drug group (Fentanyl=Ref) | 0.51 (0.15, 1.59) | 0.60 (0.08, 1.32) | 0.64 (0.11, 1.36) | 0.60 (0.10, 1.26) | 0.69 (0.17, 1.41) |

Table S5: Influence of priors on adjusted logistic regression results for each package and software not presented in the main results.

|  | MLE  (non-Bayesian) | Vague  N(0, 10000) | Skeptical  N(0, 100) | Optimistic  N(-30, 100) | Pseudo-Vague N(0, 1000) |
| --- | --- | --- | --- | --- | --- |
|  | **OR (95% CI)** | **Posterior Mean OR (95% HPD Credible Interval)** | | | |
|  | SAS - PROC MCMC | | | | |
| Intercept | 0.86 (0.33, 2.24) | 0.93 (0.20, 1.84) | 0.92 (0.21, 1.76) | 0.93 (0.27, 1.82) | 0.79 (0.25, 1.44) |
| Drug group (Fentanyl=Ref) | 0.59 (0.16, 2.16) | 0.74 (0.09, 1.75) | 0.75 (0.13, 1.67) | 0.68 (0.13, 1.51) | 0.72 (0.15, 1.51) |
| Sex  (Male = Ref) | 0.18 (0.05, 0.66) | 0.21 (0.03, 0.49) | 0.21 (0.02, 0.51) | 0.20 (0.02, 0.46) | 0.29 (0.06, 0.61) |
| Procedure Length | 1.00 (0.99, 1.02) | 1.00 (0.99, 1.02) | 1.00 (0.99, 1.02) | 1.00 (0.99, 1.02) | 1.00 (0.99, 1.02) |
|  | STATA | | | | |
| Intercept | 0.86 (0.33, 2.25) | 0.94 (0.21, 1.91) | 0.90 (0.24, 1.79) | 0.94 (0.23, 1.89) | 0.77 (0.22, 1.42) |
| Drug group (Fentanyl=Ref) | 0.59 (0.16, 2.16) | 0.71 (0.07, 1.68) | 0.75 (0.08, 1.71) | 0.68 (0.07, 1.52) | 0.74 (0.15, 1.57) |
| Sex  (Male = Ref) | 0.18 (0.05, 0.66) | 0.20 (0.02, 0.50) | 0.21 (0.02, 0.50) | 0.21 (0.03, 0.49) | 0.31 (0.06, 0.67) |
| Procedure Length | 1.00 (0.99, 1.02) | 1.00 (0.99, 1.01) | 1.00 (0.99, 1.02) | 1.00 (0.99, 1.02) | 1.00 (0.99, 1.01) |
|  | RSTAN | | | | |
| Intercept | 0.86 (0.33, 2.26) | 0.95 (0.21, 1.91) | 0.91 (0.22, 1.80) | 0.94 (0.21, 1.86) | 0.79 (0.22, 1.51) |
| Drug group (Fentanyl=Ref) | 0.59 (0.16, 2.14) | 0.72 (0.08, 1.74) | 0.76 (0.10, 1.73) | 0.70 (0.10, 1.60) | 0.80 (0.16, 1.67) |
| Sex  (Male = Ref) | 0.18 (0.04, 0.62) | 0.21 (0.02, 0.50) | 0.21 (0.02, 0.50) | 0.21 (0.02, 0.49) | 0.34 (0.07, 0.69) |
| Procedure Length | 1.00 (0.99, 1.02) | 1.00 (0.99, 1.02) | 1.00 (0.99, 1.02) | 1.00 (0.99, 1.02) | 1.00 (0.99, 1.02) |

**Diagnostic Plots of Unadjusted Analyses (representative selection)**

**Figure S1**: SAS, Unadjusted linear regression (PROC MCMC), Vague Prior N(0, 10000).


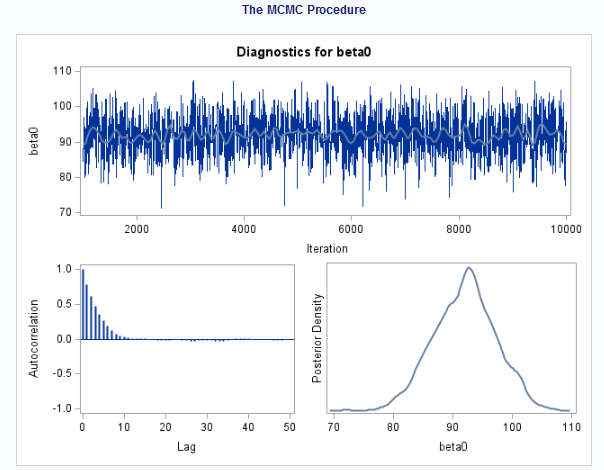

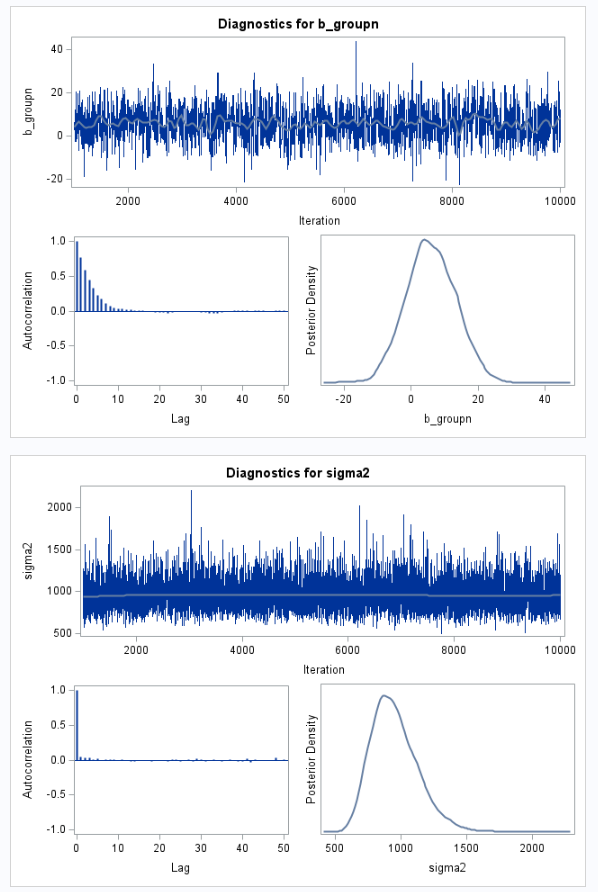


**Figure S2**: RStan, Unadjusted linear regression, Vague Prior N(0, 10000).


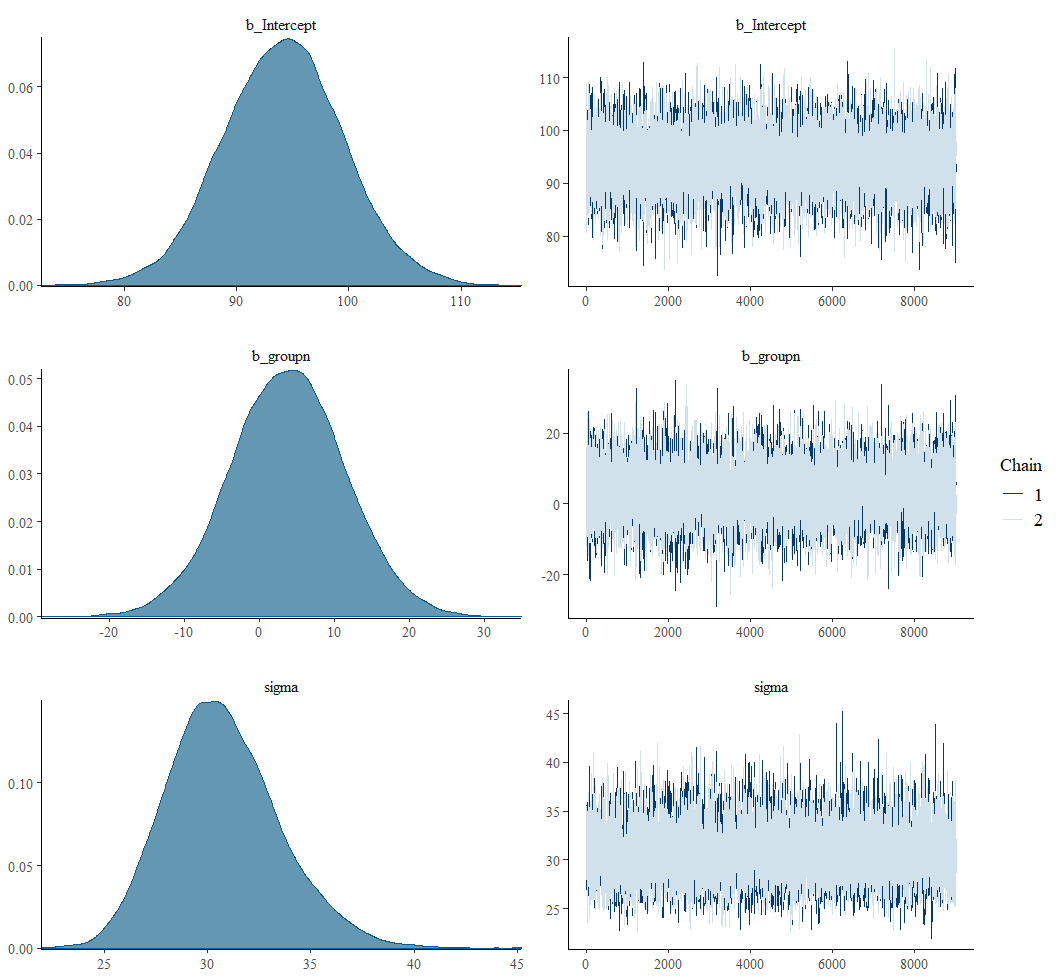


**Figure S3**: STATA, Unadjusted linear regression, Vague Prior N(0, 10000).

**Unadjusted Logistic Regression**

**Figure S4**: SAS, Unadjusted logistic regression (PROC MCMC), Vague Prior N(0, 10).


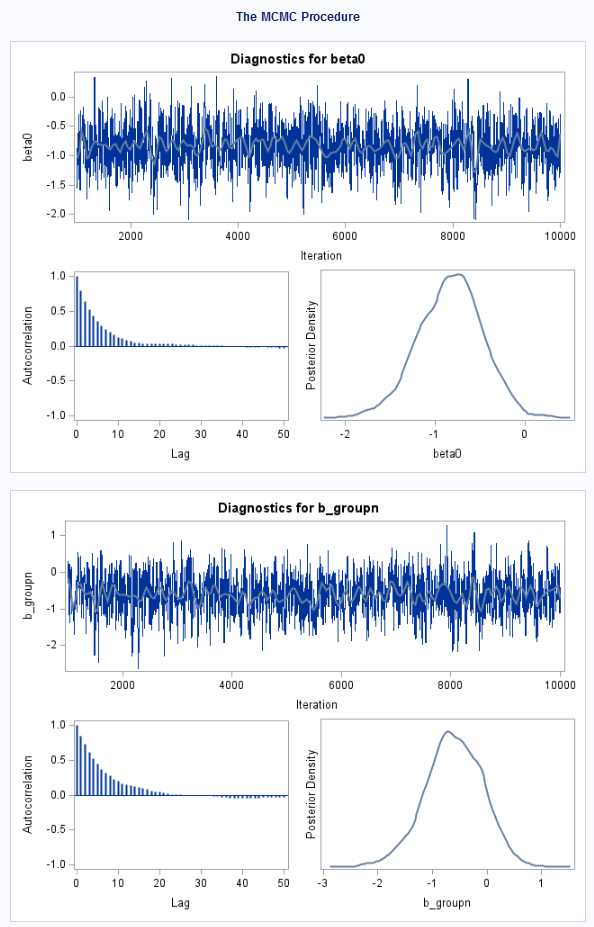


**Figure S5**: RStan, Unadjusted logistic regression, Vague Prior N(0, 10).


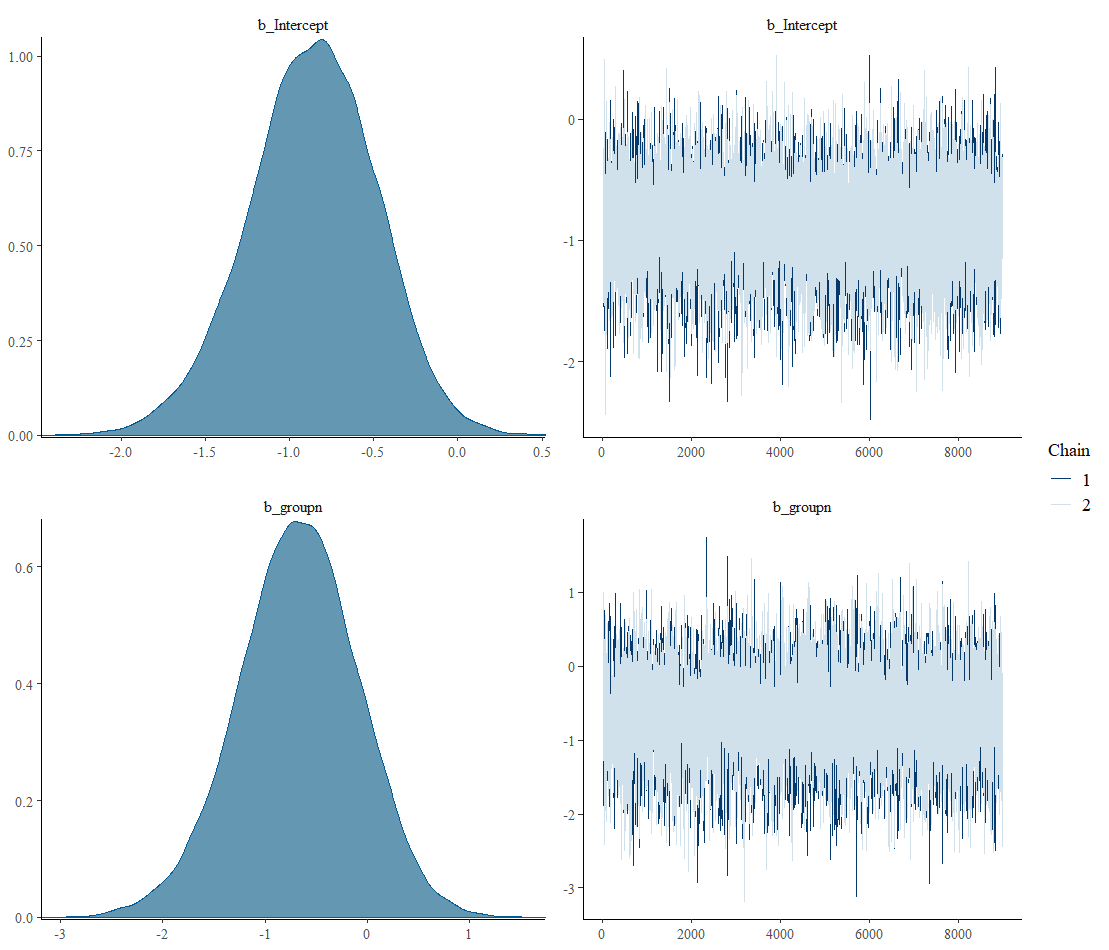


**Figure S6**: STATA, Unadjusted logistic regression, Vague Prior N(0, 10).

**Diagnostic Plots of Adjusted Analyses (representative selection)**

**Figure S7**. SAS, Adjusted linear regression, vague prior N(0,10000) for variance parameter.


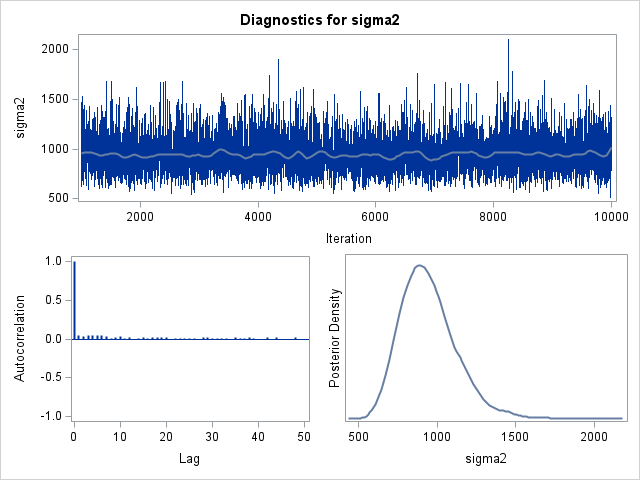


**Figure S8**. STATA, Adjusted linear regression, optimistic informative prior N(-30,100) for drug group term and vague prior N(0,10000) for intercept parameter.

**Figure S9**. R, Adjusted linear regression, autocorrelation plot, vague prior N(0,10000) for all parameters.


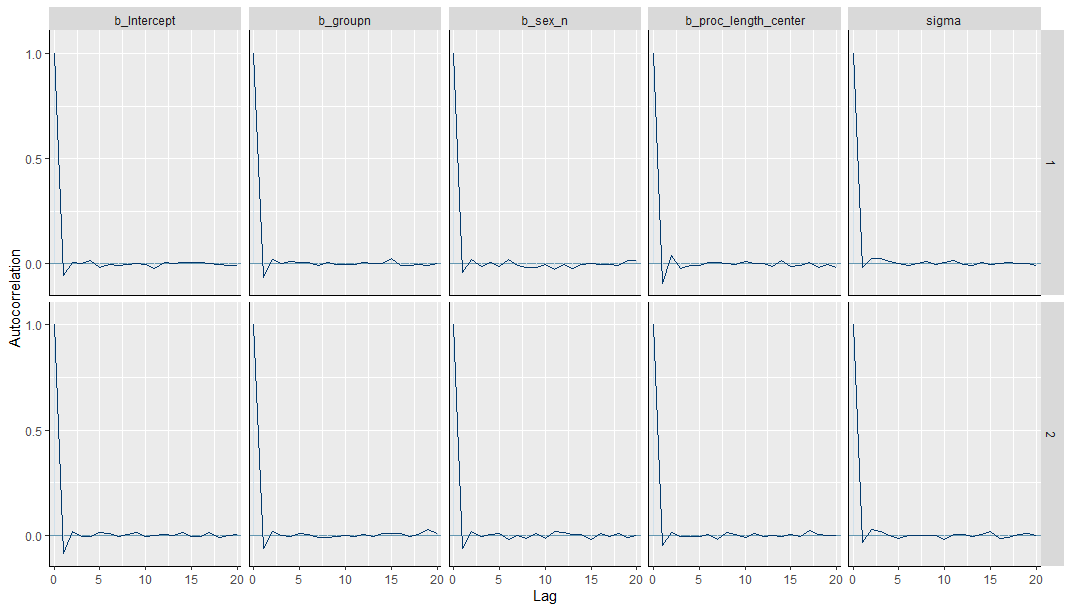


**Figure S10**. STATA, Adjusted logistic regression, skeptical informative prior N(0,2) for drug group and prior N(0,10) for sex parameter.

**Figure S11**. SAS, Adjusted logistic regression, pseudo vague prior N(0,1) for sex parameter, as odds ratio.


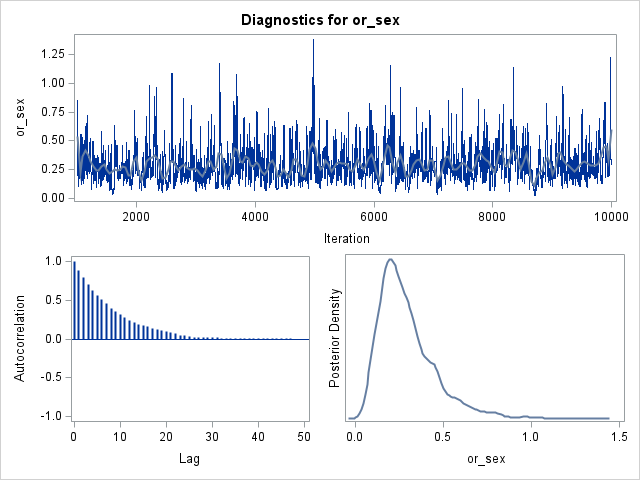


**Figure S12**. R, Adjusted logistic regression, histogram plots, pseudo vague prior N(0,1) for all parameters.


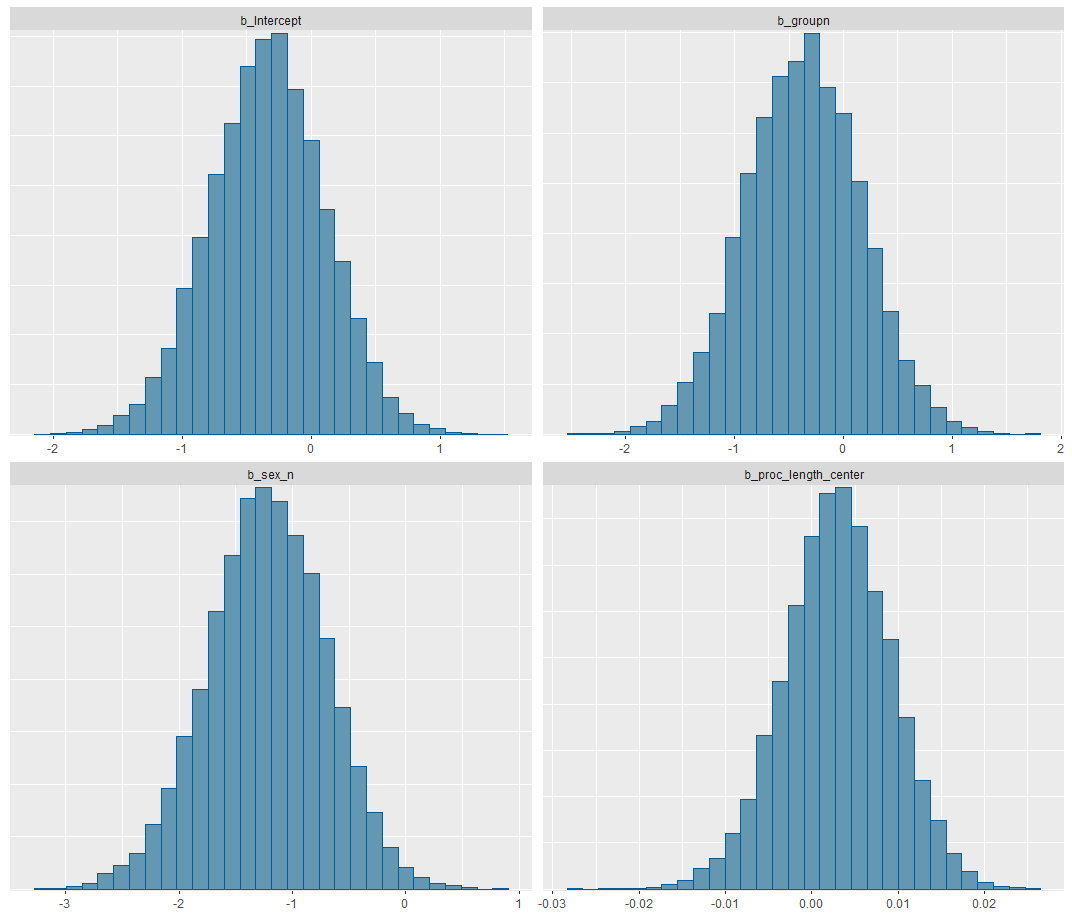

Supplement: Gunn-Sandell et al. supplementary material [file S2059866123006891sup001.docx]
